# Association between the prognostic nutritional index and early mortality of AML patients after allogeneic HSCT: a retrospective cohort analysis

**DOI:** 10.3389/fonc.2026.1754463

**Published:** 2026-02-04

**Authors:** Jie Tang, Feng Ran, Gangping Li, LiQuan Jiang, Li Wang, Yali Wang, Yimei Feng, Xiaoli Chen, Xi Zhang

**Affiliations:** 1 Department of Hematology, The Second Affiliated Hospital of Army Medical University, Chongqing, China; 2 Department of Hematology, The Affiliated Cancer Hospital of Zhengzhou University & Henan Cancer Hospital, Zhengzhou, China

**Keywords:** acute myeloid leukemia, hematopoietic stem cell transplantation, mortality, nutritional status, prognostic nutritional index

## Abstract

**Background:**

Allogeneic hematopoietic stem cell transplantation (Allo-HSCT) is an effective treatment for Acute Myeloid Leukemia (AML). The pre-transplantation Prognostic Nutritional Index (PNI) plays a critical role in determining patient prognosis, however, its correlation with mortality within 180 days after SCT is currently unclear.

**Objective:**

This study aimed to investigate the association between PNI and mortality within 180 days among AML patients who underwent HSCT.

**Methods:**

We conducted a cohort study by retrospectively collecting data from AML patients who underwent HSCT. The PNI was calculated and recorded as serum albumin (g/L) + 5 × peripheral blood lymphocyte count (10^9^/L) using pre-transplantation laboratory data. Patients were divided into three groups based on PNI tertiles. A Cox proportional hazards model was employed to explore the nonlinear relationship between PNI and mortality in these patients. The correlation between pre-transplant PNI and hematopoietic reconstitution, as well as complications such as gastrointestinal bleeding, fever, and graft-versus-host disease (GVHD), was also analyzed.

**Results:**

This retrospective cohort study included 477 adult patients with AML. The mean age of the patients was 37.4 ± 11.8 years, with males accounting for 55.6%. The vast majority (79.5%) of patients received transplants from related donors. Among those patients, 13.2% died within 180 days after transplantation. Patients with PNI T1 (30.2–44.6) had a significantly higher adjusted hazard ratio (HR) for mortality (1.52; 95% confidence intervals (CI), 1.17–1.98; *p* = 0.002) compared with PNI T2 (44.7–49.1), while PNI T3 (49.2–102.6) did not show increased risk (HR, 1.05; 95% CI, 0.81–1.35; *p* = 0.732). A PNI threshold of 46 marked a significant decrease in mortality risk with increasing PNI values, suggesting a critical point for nutritional intervention. Above this threshold, PNI showed no further prognostic value, indicating a plateau effect. These findings underscore PNI’s potential in guiding targeted nutritional support to improve post-transplant outcomes.

**Conclusions:**

The PNI exhibits a L-shaped association with 180-day mortality in AML patients post-HSCT, emerging as a significant predictor with a critical threshold identified at PNI 46. Below this threshold, declining PNI values correlate with increased mortality risk, underscoring its clinical utility in driving nutritional interventions to enhance post-transplant survival outcomes.

## 1 Introduction

Allogeneic hematopoietic stem cell transplantation (allo-HSCT) is a critical therapy for acute myeloid leukemia (AML) and other hematological malignancies (1, 2), offering long-term survival potential. However, patient prognosis after transplantation is influenced by multiple factors: the pre-transplant disease status (3), transplant conditional regimen (4), relapse prevention, and malnutrition before transplantation (5, 6). Evidence suggests that malnutrition may increase the risk of post-transplant complications, including oral mucositis (OM), acute graft-versus-host disease (GVHD), infections, and re-hospitalization rates (7, 8).

For this reason, the Global Leadership Initiative on Malnutrition (GLIM) criteria, which demonstrate prognostic value across various cancer types, are regarded as a potential assessment tool (7, 8). However, a standardized screening tool for malnutrition in the AML patient cohort undergoing HSCT is yet to be established. Prognostic Nutritional Index (PNI) is a composite index reflecting measure of nutritional and immune status, based on serum albumin levels and peripheral blood lymphocytes (9). It is a predictive factor for various diseases, reflecting nutritional status, inflammatory conditions, and overall prognosis (10). Preoperative PNI has been identified as a predictor of postoperative acute kidney injury, postoperative pneumonia in patients with intracerebral hemorrhage (11), and surgical outcomes in patients undergoing open hepatectomy for hepatocellular carcinoma (12). Within hematological diseases, PNI also has shown predictive value for outcomes in acute graft-versus-host disease (aGVHD) following allo-HSCT (13), as well as in lymphomas such as diffuse large B-cell lymphoma (DLBCL) (14), angioimmunoblastic T-cell lymphoma (15) and in primary central nervous system Lymphoma (16) etc. There is limited direct research on the relationship between PNI and childhood tumors or hematologic malignancies. However, some studies suggest that PNI may influence the prognosis of hematologic malignancies (such as multiple myeloma) through nutritional and immune status (17), and it holds potential value in nutritional risk assessment for children (18). Furthermore, PNI has been linked to both short-term and long-term survival in cancer patients (19).

However, the prognostic value of the PNI in predicting survival outcomes for AML patients undergoing HSCT remains uncertain. This study aimed to evaluate the association between PNI and mortality within 180 days in AML patients receiving their first HSCT.

## 2 Materials and methods

### 2.1 Study population

**Figure 1:**
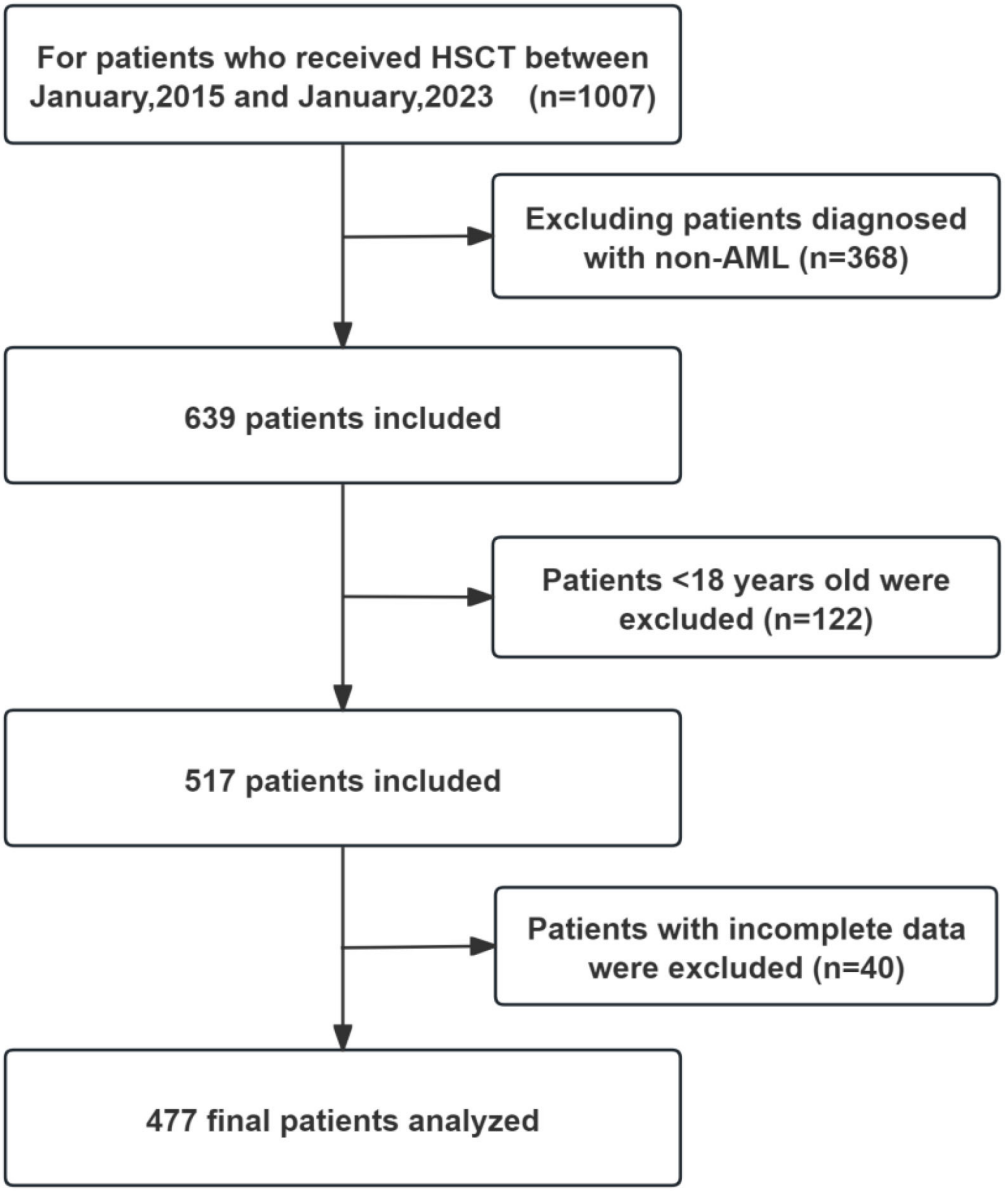
Flow chart of this research.

Patients were eligible for inclusion if they were: ① aged between 18 and 70 years; ② diagnosed with AML; ③ candidates for first allogeneic HSCT between January 2015 and December 2021. Exclusion criteria were: ① prior allogeneic or autologous HSCT; ② active concurrent malignancy; ③ incomplete baseline laboratory data for PNI calculation; ④ loss to follow-up within 180 days post-HSCT. A total of 477 adult patients underwent Allo-HSCT were monitored from the initial assessment prior to transplantation until 180 days post-transplant. During this period, outcomes were studied through the medical records of the patients (**Figure 1**).

### 2.2 Primary and secondary endpoints

The primary endpoint was all-cause mortality within 180 days post-HSCT. Secondary endpoints included: ① time to hematopoietic reconstitution, defined as the first of three consecutive days with an absolute neutrophil count (ANC) ≥ 0.5 × 10^9^/L and a platelet count ≥ 20 × 10^9^/L without transfusion support in the preceding 7 days (20); ② the incidence of acute GVHD, diagnosed and graded according to the Glucksberg criteria (21); ③ the incidence of documented bacterial or fungal infections within 180 days; ④ Overall survival (OS) was defined as the time from stem cell infusion to death from any cause; ⑤ Event-free survival (EFS) was defined as the time from stem cell infusion to disease relapse/progression or death (22).

### 2.3 Assessment of PNI and categorization

In this study, we systematically retrieved patient data from the electronic medical records. We collected demographic, clinical, and laboratory data (complete blood counts and biochemical profiles), including comorbidities and mortality details. Additional variables encompassed age, sex, and transplant-specific details, alongside 180-day mortality and hematopoietic recovery times. All baseline data were gathered within 48 hours post-admission to the transplant ward. The PNI was determined using the formula: PNI = Albumin (g/L) + 5 × ALC (Absolute lymphocyte count, ×10^9^/L) (12).

To stratify clinical risk, patients were categorized into three groups (T1, T2, T3) based on tertile divisions of PNI values within this cohort, corresponding to low-risk, intermediate-risk, and high-risk classifications. Our patients were divided into three groups: T1 (30.2-44.6), T2 (44.7-49.1), and T3 (49.2-102.6), to explore the relationship between pre-transplant PNI and 180-day mortality. This classification method aligns with the commonly used tertile-based stratification strategy in prognostic biomarker studies (13, 23), which helps identify different risk subgroups and provides a practical framework for clinical risk stratification (24, 25).

### 2.4 Conditioning regimen and post-transplant complication management

The Conditioning regimens in this study are primarily categorized into myeloablative conditioning (MAC) and non-myeloablative conditioning (NMAC) regimens. The MAC regimens consisted of busulfan (BU 3.2 mg/kg/day, − 6 to -4 day) + cyclophosphamide (CY 50 mg/kg/day, − 3 to -2 day) for identical transplantation, and chloroethyl-cyclohexyl-nitrosourea (CCNU, 200 mg/m^2^, − 9 day) + BU(3.2 mg/kg/day, − 6 to -4 day) + CY(1.8 g/m^2^/day, − 3 to -2 day) + Cytarabine (Ara-C 6 g/m^2^/day, − 5 to − 3 day) + Antithymocyte globulin (ATG 2.5 mg/kg/day − 5 to -2 day) for haploidentical transplantation. The NMAC, commonly using a regimen of fludarabine (30 mg/m²/day for 4–5 days) combined with busulfan (3.2 mg/kg/day for 2–3 days) (22). The acute GVHD prophylaxis regimen included tacrolimus or cyclosporine A, methotrexate, and low-dose, short-course mycophenolate mofetil (26). The management of chronic GVHD refers to the consensus of Chinese experts (27).

Transplant-related mortality (TRM) refers to the death rate of patients who die from complications directly related to the hematopoietic stem cell transplantation process itself, rather than from relapse or progression of the original disease. Transplant-Related Complications (TRCs) are a critical concern in HSCT, broadly categorized by their timing and underlying pathophysiology. Early TRCs Originating from Endothelial Damage. These complications typically occur within the first 100 days post-transplant and are driven primarily by endothelial injury triggered by high-dose chemotherapy, radiation, immunosuppressive agents, or infections. Endothelial dysfunction leads to a cascade of inflammatory and thrombotic events, resulting in: Sinusoidal Obstruction Syndrome/Veno-Occlusive Disease (SOS/VOD), Capillary Leak Syndrome (CLS), Engraftment Syndrome (ES), and Transplant-Associated Thrombotic Microangiopathy (TA-TMA) (28). Late TRCs emerge beyond 100 days post-transplant and often result from prolonged immune dysregulation, chronic inflammation, or the delayed effects of pre-transplant conditioning. Key late TRCs include: Endocrine Dysfunction, Musculoskeletal Disorders, Secondary Malignancies, and Other Chronic Organ Toxicities. The management of the above complications are based on the EBMT handbook (29), and BMT handbook (30).

### 2.5 Sample size calculation and statistical analysis

For sample size calculation, we conducted a *post hoc* power analysis using PASS 2021 software. Considering a 10% dropout rate, two-sided α=0.05, 90% power, and the observed 180-day mortality rate (13.2%), the current sample size was sufficient to detect a hazard ratio (HR) ≥1.52 between the lowest PNI tertile group and the middle group. The estimated required sample size was approximately 400–450 cases, indicating that the sample size in this study was adequate.

Continuous variable data were described as mean ± standard deviation (SD) or median and interquartile range (IQR), while categorical variable data were described as frequency or percentage. For the analysis of baseline characteristics, comparisons of data were made using the Mann-Whitney test for continuous variables and the chi-square test for categorical variables. Univariate and multivariate Cox regression analyses, employing unadjusted and multivariable-adjusted models, were used to determine the stability of the relationship. All analyses were performed using the statistical software packages R 3.3.2 (http://www.R-project.org, The R Foundation, Shanghai, China) and Free Statistics software version 1.9.2. A descriptive study was conducted on all participants. All comparisons were planned, two-tailed tests were performed, and *p* < 0.05 was considered statistically significant, indicating a significant difference between two or more groups.

Ethical approval for this study was obtained from the Ethics Committee of the Second Affiliated Hospital of Army Medical University (2023-study-146-01). Patient data were anonymized before analysis, thus waiving the need for written or verbal consent. All procedures were conducted in accordance with the Declaration of Helsinki.

## 3 Results

### 3.1 Baseline characteristics

The characteristics of the study population based on PNI Quartiles was summarized in **Table 1**. 477 patients met the inclusion criteria and were enrolled. The average age of patients was 37.1 (27.0, 47.0) years, male was 56.0% and female 44%. Among these, 227 cases (47.6%) received HLA matched HSCT, while 250 cases (52.4%) were haplo-HSCT. Out of 477 cases, 379 (79.5%) received transplants from related donors, while 98 (20.5%) received transplants from unrelated donors. The average count of infused CD34^+^ cells was 6.0 (4.3, 7.5) ×10^6^/kg (**Table 1**).

**Table 1:** Baseline characteristics of the study population.

Variables	Prognostic nutritional index quartiles				*P*
	Total (n = 477)	T1 (30.2–44.6)	T2 (44.7–49.1)	T3 (49.2–102.6)	
Patient sex, n (%)					0.001
Male	265 (55.6)	70 (44)	93 (58.9)	102 (63.7)	
Female	212 (44.4)	89 (56)	65 (41.1)	58 (36.2)	
Patient age(Mean± SD)	37.4 ± 11.8	40.1 ± 11.8	36.7 ± 11.7	35.5 ± 11.6	0.002
History of Cancer, n (%)					0.25
No	389 (83.1)	125 (79.1)	131 (85.6)	133 (84.7)	
Yes	79 (16.9)	33 (20.9)	22 (14.4)	24 (15.3)	
BMI, Mean ± SD	22.1 ± 3.6	21.9 ± 3.6	22.4 ± 3.9	22.1 ± 3.2	0.574
Risk group, n (%)					0.07
Low	20 (4.2)	9 (5.7)	4 (2.5)	7 (4.4)	
Middle	130 (27.3)	31 (19.5)	51 (32.3)	48 (30)	
High	327 (68.6)	119 (74.8)	103 (65.2)	105 (65.6)	
Chemotherapy≥3 cycles before HSCT, n (%)					0.361
No	58 (12.2)	20 (12.6)	23 (14.6)	15 (9.4)	
Yes	419 (87.8)	139 (87.4)	135 (85.4)	145 (90.6)	
HLA match, n (%)					0.029
Identical	227 (47.6)	89 (56)	66 (41.8)	72 (45)	
Non-identical	250 (52.4)	70 (44)	92 (58.2)	88 (55)	
Doner type, n (%)					0.023
Related	379 (79.5)	115 (72.3)	130 (82.3)	134 (83.8)	
Unrelated	98 (20.5)	44 (27.7)	28 (17.7)	26 (16.2)	
Donor sex, n (%)					0.35
Male	348 (73.9)	124 (78)	113 (72)	111 (71.6)	
Female	123 (26.1)	35 (22)	44 (28)	44 (28.4)	
Disease Status, n (%)					0.046
Alive	398 (83.4)	126 (79.2)	141 (89.2)	131 (81.9)	
Death	79 (16.6)	33 (20.8)	17 (10.8)	29 (18.1)	
HC, n (%)					0.242
No	441 (92.5)	143 (89.9)	150 (94.9)	148 (92.5)	
Yes	36 (7.5)	16 (10.1)	8 (5.1)	12 (7.5)	
Infection, n (%)					0.229
No	334 (70.0)	106 (66.7)	108 (68.4)	120 (75)	
Yes	143 (30.0)	53 (33.3)	50 (31.6)	40 (25)	
OM, n (%)					0.041
No	381 (79.9)	120 (75.5)	123 (77.8)	138 (86.2)	
Yes	96 (20.1)	39 (24.5)	35 (22.2)	22 (13.8)	
GVHD, n (%)					0.981
No	255 (53.5)	86 (54.1)	84 (53.2)	85 (53.1)	
Yes	222 (46.5)	73 (45.9)	74 (46.8)	75 (46.9)	
Infused CD34^+^, ×10^6^/kg	6.0 (4.3, 7.5)	6.1 (4.4, 7.9)	6.1 (4.5, 7.3)	5.8 (4.1, 7.3)	0.286

BMI, Body mass index; HLA, Human Leukocyte Antigen; HSCT, Hematopoietic Stem Cell Transplantation; HC, Hemorrhagic cystitis; OM, oral mucositis; GVHD, Graft-versus-host disease.

### 3.2 Associations between PNI and transplant outcomes

During the follow-up period until 180 days post-HSCT, a total of 63 patients (13.2%) died. After multivariate adjustment for age, sex, BMI, HLA matching, disease risk stratification, donor sex, infused CD34^+^ cell count, hemoglobin, white blood cell count, neutrophils, and platelets, in Model 2, the first quartile showed a significant association with 180-day PNI and mortality compared to the reference second quartile. Further adjustment for potential confounding factors yielded consistent results across other models. Model 1 did not adjust for covariates. Model 2 was adjusted for age, sex, BMI, and HLA matching. Model 3 was adjusted for the variables in Model 2 plus donor sex, CD34^+^ cells, and hemoglobin. In the fully adjusted Model 4, patients undergoing HSCT were categorized into three groups based on their PNI scores. Model 4 adjusted for all covariates, the HR for mortality at 180 days for the lowest PNI score group, T1 (30.2–44.6), was 1.52 (95% *CI*, 1.17–1.98; *p* = 0.002), indicating that patients in the low PNI group had a 52% higher risk of death compared to those in the normal PNI group. Conversely, the risk for the highest PNI group, T3 (49.2–102.6), did not show a significant increase (HR, 1.05; 95% *CI*, 0.81–1.35; *p* = 0.732) (**Table 2**).

**Table 2:** Multivariable Cox regression analyses for 180-day mortality in patients with HSCT.

Variable	N (total)	Model 1		Model 2		Model 3		Model 4	
		HR (95%CI)	P value	HR (95%CI)	P value	HR (95%CI)	P value	HR (95%CI)	P value
PNI									
T1 (30.2–44.6)	158	1.45 (1.14~1.84)	0.003	1.45 (1.12~1.86)	0.004	1.47 (1.13~1.9)	0.004	1.52 (1.17~1.98)	0.002
T2 (44.7–49.1)	159	1(Ref)		1(Ref)		1(Ref)		1(Ref)	
T3(49.2–102.6)	160	1.09 (0.86~1.37)	0.491	1.09 (0.86~1.38)	0.487	1.04 (0.81~1.33)	0.78	1.05 (0.81~1.35)	0.732

Model 1: No covariates were adjusted.

Model 2: Adjusted for age, sex, BMI, HLA match, Risk.

Model 3: Adjusted for Model2 and Doner sex, CD34^+^ cell, haemoglobin.

Model 4: Adjusted for Model3 and White blood cell, Neutrophil, Platelet.

### 3.3 Low PNI delayed the hematopoietic engraftment

In our study, the risk of delayed platelet hematopoietic engraftment was 39% higher than the control group (T1) (HR, 1.39; 95% *CI*, 1.08-1.78; *p* = 0.01). There was no statistically significant difference in risk in the group with the highest PNI (T3) (HR, 1.05; 95% *CI*, 0.83-1.33; *p* = 0.659). The study suggests that a low PNI score before transplantation indicates a slower recovery of hematopoietic function after HSCT, emphasizing the importance of nutritional status (**Table 3**).

**Table 3:** Association between PNI and DNE/DPE after HSCT by multivariable analyses.

Variable	N (total)	DNE		DPE	
		HR (95%CI)	P value	HR (95%CI)	P value
PNI					
T1 (30.2–44.6)	158	1.39 (1.09~1.77)	0.008	1.39 (1.08~1.78)	0.01
T2 (44.7–49.1)	159	1(Ref)		1(Ref)	
T3(49.2–102.6)	160	1.05 (0.84~1.33)	0.663	1.05 (0.83~1.33)	0.659

Model: Adjusted for age, sex, HLA match, Risk, Doner sex, CD34^+^ cell.

DNE, Delayed Neutrophil Engraftment; DPE, Delayed Platelet Engraftment.

### 3.4 Threshold effect analysis of PNI on mortality within 180 days

**Figure 2:**
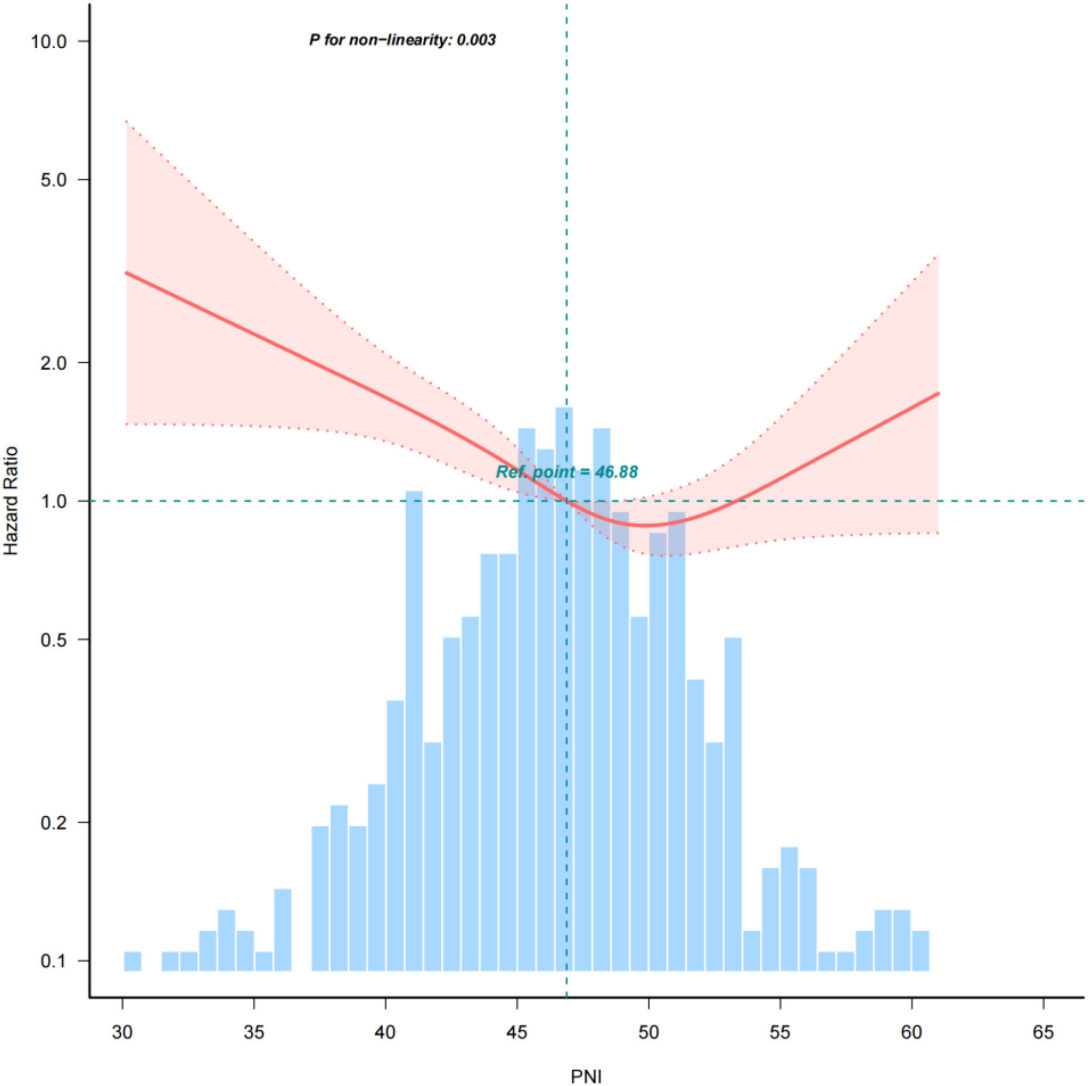
Relationship between PNI and mortality within 180 days after transplantation The analysis identifies a nonlinear relationship between pre-transplant PNI and early mortality (P for nonlinearity = 0.003), indicating a dynamic risk association rather than a simple linear trend. The inflection point at a PNI of 46.88 (red dot) suggests a critical threshold for nutritional risk stratification.

A multivariate Cox regression model, complemented by smooth curve fitting, was employed to elucidate the relationship between the PNI and 180-day mortality. The analysis revealed a distinct L-shaped curve (nonlinear, *p* = 0.003) between PNI and mortality risk within 180 days post-HSCT, as depicted in **Figure 2**. This curve suggests a threshold effect, with PNI values exerting a significant impact on mortality below a critical threshold. Specifically, the hazard ratio (HR) for 180-day mortality among participants with PNI less than 46 was 0.93 (95% *CI*, 0.88–0.98; *p* = 0.003), indicating a 7.2% reduction in mortality for each 1-unit increase in PNI. In contrast, when the PNI was greater than or equal to 46, there was no significant change in 180-day mortality (**Table 4**). As far as we know, this is the first report to analyze and evaluate the correlation between PNI and the mortality rate of AML patients after transplantation.

**Table 4:** The non-linearity relationship between PNI and 180-day mortality.

PNI	HR	95%CI	P value
<46	0.93	0.883,0.98	0.003
≥46	1.03	0.98,1.08	0.270
Non-linear test			0.003

Adjusted for sex, age, risk, HLA match, doner sex, infused CD34^+^.

### 3.5 Association between pre-transplant PNI and other complications after HSCT

Multivariate analysis indicated that pre-transplant PNI showed no correlation with post-transplant complications such as fever, acute GVHD, hemorrhagic cystitis (HC), or oral mucositis (OM) (p>0.05). However, a lower pre-transplant PNI was associated with an increased risk of gastrointestinal bleeding after transplantation. In other words, for each unit increase in PNI, the risk of gastrointestinal bleeding decreased by 7% (*p* = 0.041) (**Table 5**).

**Table 5:** Association between pre-transplant PNI and other complications after HSCT.

Variable	Event (%)	Univariate analyses		Multivariate analyses	
		OR (95%CI)	P value	OR (95%CI)	P value
Fever	143 (30)	0.97 (0.94~1.01)	0.094	0.98 (0.94~1.01)	0.166
aGVHD	222 (46.5)	1 (0.97~1.03)	0.85	0.99 (0.96~1.02)	0.44
GIB	35 (7.3)	0.93 (0.87~0.99)	0.019	0.93 (0.87~1)	0.041
HC	36 (7.5)	0.96 (0.9~1.02)	0.167	0.96 (0.89~1.02)	0.19
OM	96 (20.1)	0.96 (0.92~1)	0.061	0.96 (0.92~1)	0.07

aGVHD, acute Graft-versus-host disease; GIB, Gastrointestinal Bleeding; HC, Hemorrhagic cystitis; OM, Oral Mucositis.

### 3.6 Subgroup analysis

**Figure 3:**
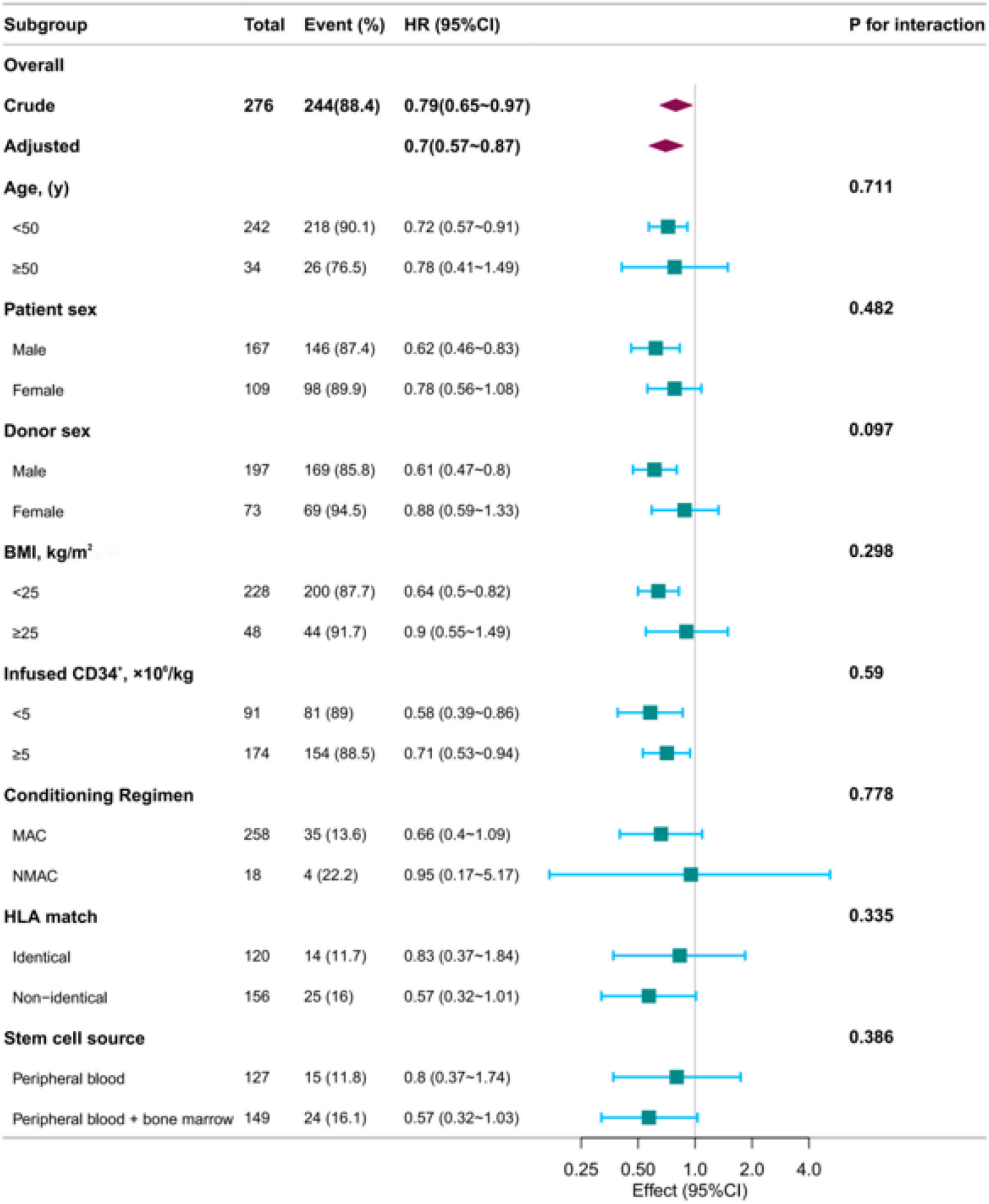
Subgroup analysis association between PNI and mortality within 180 days after transplantation. Forest plot illustrates the consistent protective association of higher PNI with reduced mortality within 180 days after transplantation in most patient subgroups. Hazard ratios and 95% confidence intervals are derived from multivariable-adjusted models. The P for interaction values indicate no significant effect modification by age, sex, donor sex, platelet count, BMI, or infused CD34^+^ cell dose.

Our study’s subgroup analyses, which assessed the relationship between the PNI and 180-day mortality across various demographic and treatment-related factors such as age, sex, donor sex, BMI, and infused CD34^+^ counts, conditioning regimen, HLA matching degree, and stem cell source, confirmed a significant and consistent correlation with the overall study findings (**Figure 3**). The uniformity of these findings across subgroups underscores the robust prognostic value of PNI, which is not significantly confounded by patient demographics or treatment specifics. The absence of significant interactions in any subgroup further substantiates the broad applicability of PNI as a reliable predictor of early post-transplant mortality, highlighting its potential role in clinical decision-making and personalized patient care strategies to optimize survival outcomes in AML patients undergoing HSCT.

## 4 Discussion

Research on nutritional status during Allo-HSCT has revealed that malnutrition is a critical factor impacting patient outcomes (31). The European and American Nutrition Society guidelines emphasize the importance of nutritional assessment for allogeneic transplant patients to identify the need for nutritional support (7). PNI is key indicator for evaluating nutritional status (32).Serum albumin levels are a reliable indicator for assessing disease severity and prognosis (33, 34). Lymphocyte count is a biomarker that reflects immune status and is associated with the proliferation, invasion, and migration of cancer cells (35). Low lymphocyte count not only indicates impaired immune function, but is also associated with malnutrition (36). Hypoalbuminemia is often associated with inflammation and protein loss, while malnutrition can delay immune system reconstruction after HSCT and increase the risk of infection (37). The decrease in PNI may reflect a chronic inflammatory state and be associated with adverse clinical outcomes after HSCT (13).

In clinical practice, monitoring key indicators such as serum albumin and lymphocyte count is crucial for assessing patient nutritional status. The PNI, which combines these indicators, provides a convenient method to evaluate both nutritional and immune status, demonstrating significant prognostic value for patients with hematological malignancies (35, 38). Our study revealed a non-linear relationship between PNI and 180-day mortality through Cox regression analysis, consistent with findings in DLBCL patients (39). These results are consistent with the findings (40), confirming that the PNI serves as an independent prognostic factor in AML patients following transplantation. Recent meta-analyses further support the positive correlation between PNI and overall patient survival (41). Due to its ease of measurement and strong association with short-term mortality, PNI has proven valuable for clinical monitoring.

Certainly, the death of AML patients within 180 days post-transplant can be attributed to multiple factors, including bleeding, infection, GVHD, disease relapse, and malnutrition. In our study, we did not claim that PNI is the sole factor influencing early mortality but rather identified its significant correlation with 180-day mortality. A PNI below 46 may increase the risk of death within this period. Additionally, PNI has been shown to significantly correlate with in-hospital postoperative mortality and 1-year all-cause mortality in cardiac surgery patients (42). Similarly, in elderly burn patients, PNI has been evaluated as a predictor of one-year mortality (43). Based on our findings and existing research, we have reasonable grounds to conclude that PNI is associated with 180-day mortality in AML patients after transplantation. However, as this is a retrospective study, further validation through multicenter, large-scale studies is necessary.

We acknowledge that pre-transplant nutritional status may not be the sole factor influencing post-transplant mortality, but this study identifies it as a significant contributor. The potential mechanisms include: high-intensity conditioning regimens almost inevitably lead to post-transplant complications such as mucositis (severe gastrointestinal inflammation), nausea, vomiting, anorexia, and malabsorption in patients, resulting in rapid and significant weight loss and deterioration of nutritional status. This may subsequently exacerbate late-phase endothelial injury syndromes (VOD, TMA, GVHD, etc.), impair patients’ quality of life, and more likely increase early mortality (44). We performed a multivariate analysis of patient baseline characteristics and 180-day mortality. The results showed that patients receiving non-identical transplants had a 180-day mortality rate twice as high as those receiving identical transplants (*p* = 0.005; **Supplementary Table S1**). This disparity may be attributed to more intensive conditioning regimens and a higher incidence of late complications, such as GVHD, in non-identical transplant recipients. In fact, nearly 100% of our transplant patients received parenteral nutritional therapy during the transplantation period. Although this study is a retrospective observational analysis, its conclusion that a low pre-transplant PNI increases mortality within 180 days post-transplant, which strongly suggests the need to improve pre-transplant PNI to improve outcomes. For instance, increasing nutritional intake to raise PNI above 46 could be a viable strategy.

Additionally, combining nutritional assessment with prognostic risk evaluation may be more effective, specifically by integrating the PNI with BMI, which has been applied in disease models for both adults and children (45, 46). Although our study found no significant difference in BMI values among the T1, T2, and T3 groups (*p* = 0.547, **Table 1**), composite indicators (such as grouping classifications based on high/low PNI and high/low BMI) can provide higher predictive accuracy than single parameters, offering a more comprehensive reflection of patients’ overall risk. Therefore, in our next study, we plan to incorporate BMI, CONUT scores, NRS-2002 scores, and other metrics to develop a multimodal predictive model for nutritional risk.

It is challenging to determine whether the PNI reflects baseline nutritional status or is a direct consequence of the disease itself and its treatment. Special attention should be paid: A low PNI measured before transplantation could serve either as a direct contributing factor to poor prognosis (through malnutrition), or a surrogate marker for disease aggressiveness or systemic inflammatory state (e.g., leukemia-related cachexia) (47). The latter scenario would simultaneously lead to both decreased PNI values and increased early mortality. Secondly, since our study initially only assessed pre-transplant PNI and lacked PNI measurements at initial diagnosis and after first-line treatment, there is currently no clear standard for the optimal timing of measurement (before conditioning chemotherapy, on the day of transplantation, or through dynamic monitoring). It should also be noted that lymphocyte counts are directly influenced by the intensity of the conditioning regimen (myeloablative *vs*. reduced-intensity) (48). Thirdly, parenteral nutrition and voluntary oral intake have different effects on albumin levels, which may confound the interpretation of PNI as an inherent prognostic factor, because the nutritional support methods in the study cohort were not homogeneous.

The limitations of the research include the following points: The retrospective design may have inherent biases; The dual role of PNI in reflecting both nutritional status and disease severity was not fully considered; Potential confounding factors (such as inflammatory markers) were not fully adjusted for; And there is a lack of interventional evidence to support the clinical guidance value of PNI.

## 5 Conclusions

This study confirms that the PNI is significantly correlated with mortality within 180 days post-transplantation in patients with AML. This association remained consistent across all demographic and treatment-related subgroups, including age, sex, donor sex, BMI, and infused CD34^+^ cell counts, demonstrating the reliability of PNI as a prognostic tool. A critical threshold was identified at a PNI value of 46, below which mortality risk rose significantly. This suggests PNI could guide targeted nutritional interventions prior to transplantation.

## Data Availability

The raw data supporting the conclusions of this article will be made available by the authors, without undue reservation.
