# Supplementary material for: Association between the prognostic nutritional index and early mortality of AML patients after allogeneic HSCT: a retrospective cohort analysis
Source: Front Oncol. 2026 Feb 4;16:1754463. doi: 10.3389/fonc.2026.1754463 (PMC12913105; doi:10.3389/fonc.2026.1754463)
Supplement: Supplementary file 1 [file Table1.docx]

**Table S1. Association between baseline characteristics and 180-day mortality after HSCT**

| Variable | **Univariate analyses** | | |  | **Multivariate analyses** | | |
| --- | --- | --- | --- | --- | --- | --- | --- |
|  | HR | *95%CI* | *P* value |  | HR | *95%CI* | *P* value |
| **Conditioning regimen, n (%)** |  |  |  |  |  |  |  |
| NMAC | 1(Ref) |  |  |  | 1(Ref) |  |  |
| MAC | 0.67 | 0.32~1.39 | 0.279 |  | 0.67 | 0.3~1.52 | 0.338 |
| **HLA match, n (%)** |  |  |  |  |  |  |  |
| Identical | 1(Ref) |  |  |  | 1(Ref) |  |  |
| Non-identical | 1.75 | 1.1~2.76 | 0.017 |  | 2 | 1.23~3.26 | 0.005 |
| **Patient sex, n (%)** |  |  |  |  |  |  |  |
| Male | 1(Ref) |  |  |  | 1(Ref) |  |  |
| Female | 0.74 | 0.47~1.17 | 0.201 |  | 0.76 | 0.48~1.23 | 0.268 |
| **Patient age** | 1.01 | 0.99~1.03) | 0.195 |  | 1.02 | 1~1.04 | 0.057 |
| **History of Cancer, n (%)** |  |  |  |  |  |  |  |
| No | 1(Ref) |  |  |  | 1(Ref) |  |  |
| Yes | 1.55 | 0.86~2.8 | 0.144 |  | 1.34 | 0.7~2.58) | 0.375 |
| **BMI, Mean ± SD** | 0.98 | 0.92~1.05 | 0.621 |  | 0.97 | 0.9~1.04 | 0.352 |
| **Risk group, n (%)** |  |  |  |  |  |  |  |
| Low | 2.18 | 0.29~16.41 | 0.451 |  | 1.93 | 0.25~14.69 | 0.525 |
| Middle | 3.51 | 0.49~25.3 | 0.213 |  | 3.08 | 0.42~22.41 | 0.266 |
| High | 2.18 | 0.29~16.41 | 0.451 |  | 1.93 | 0.25~14.69 | 0.525 |
| **Chemotherapy of cycles** |  |  |  |  |  |  |  |
| ≤3 | 1(Ref) |  |  |  | 1(Ref) |  |  |
| >3 | 1.59 | 0.64~3.94 | 0.318 |  | 1.3 | 0.52~3.28 | 0.574 |
| **Doner type, n (%)** |  |  |  |  |  |  |  |
| Related | 1(Ref) |  |  |  | 1(Ref) |  |  |
| Unrelated | 1 | 0.58~1.73 | 0.997 |  | 1.86 | 0.85~4.1 | 0.122 |
| **Donor sex, n (%)** |  |  |  |  |  |  |  |
| Male | 1(Ref) |  |  |  | 1(Ref) |  |  |
| Female | 0.95 | 0.58~1.57 | 0.856 |  | 0.91 | 0.54~1.53 | 0.722 |
| **Disease status before HSCT** |  |  |  |  |  |  |  |
| CR | 1(Ref) |  |  |  | 1(Ref) |  |  |
| Non-CR | 0.67 | 0.32~1.39 | 0.279 |  | 0.67 | 0.30~1.52 | 0.338 |
| **Infused CD34^+^, ×10^6^/kg** | 1 | 0.97~1.02 | 0.744 |  | 1 | 0.97~1.02 | 0.734 |
